# Medical and Philosophical Intelligence

**Published:** 1813-04

**Authors:** 


### MEDICAL AND PHILOSOPHICAL INTELLIGENCE.

### PHILOSOPHICAL SOCIETY OF LONDON.

THE learned president, Dr. Lettsom, has, during the last month, delivered a Lecture on the Natural and Medicinal Histories of Tea. He commenced by observing, that he could not be ignorant in presenting an object of discussion that it is expected that some extent of novelty may be afforded, and some useful information conveyed; but from the universally known subject, he feared that disappointment must result, for what is familiar admits of little novelty, and what is known of little interest. "To convey instruction," said the Doctor, "I do not aspire; but impelled by a cordial impulse to evince my respect for, and approbation of, this society, as well as to excite the labors of others, I have presumed to throw the discus which a more youthful and vigorous arm is better qualified to direct."

The lecturer then proceeded upon the botanical history, and having given a description of the parts of fructification, stated that there is but one species of the tea plant, the difference of green and bohea tea depending upon the nature of the soil, the culture, and manner of drying the leaves. Sir John Hill, from observing a different number of petals in different corollas, described the green and bohea tea as different species, giving to the first nine, and to the latter only six, petals. He conveyed this opinion to Linnè, who adopted the mistake which his future experience corrected, as he informed Dr. L. by letter.

The authors who have treated upon this subject amount to at least one hundred, many of whom never saw the tea tree. The first figure given of it was in the Acta Haffniensia, which was taken from a dried specimen: since that it has been figured by Boutius, Plukenet, Kœmpfer, and Lettsom, the latter the only perfect one. He had access access to the first plant grown in Europe, which was raised by his ingenious friend Mr. Ellis, in Gray's Inn, from seeds taken out of a cannister and promiscuously sown in a pot placed outside of his window. As China and Japan are the only countries known to us where the tea shrub is cultivated for use, we may reasonably conclude that it is indigenous to one of them, if not to both, and probably the brackish ill-tasted water in many parts of these countries first led to its use as an infusion.

Tea was first introduced into Europe by the Dutch East-India Company early in the sixteenth century, and a quantity of it was brought over from Holland in 1666, by Lords Arlington and Ossory. In consequence of this, tea soon became known amongst people of fashion, and its use, by degrees, since that period, has become general. Cornelius Boutcekoe, a Dutch physician, wrote a treatise in praise of tea as early as 1678.—The lecturer passed on to consider the soil and culture best adapted to this plant, and observed, that, according to Kœmpfer, no particular gardens or fields are allotted for it, but that it is cultivated round the borders of rice and corn-fields without any regard to the soil; that there are usually from six to twelve seeds in each vessel; that they are promiscuously put into a hole four or five inches deep, at certain distances from each other. The reason why so many seeds are put into one hole, is, that they contain a great quantity of oil, which is apt to turn rancid, and then they will not germinate. They then vegetate without further care. The leaves are not fit to be plucked before the third year's growth, and, in about seven years, the shrub rises to a man's height; but, as it is then but scantily provided with leaves, it is cut down to the stem, from which an exuberance of fresh shoots arise. The tea tree delights particularly in vallies, or on the declivities of hills, and upon the banks of rivers, where it enjoys a southern exposure to the sun; though it endures considerable variations of heat and cold, as it flourishes in the northern clime of Pekin, as well as about Canton.

The doctor then proceeded to describe the manner and the seasons of gathering the leaves, and the method of curing or preparing tea in Japan. Of the varieties of tea Dr. L. observed, of the green, the bing, imperial, or bloom, tea; the hy-tiann, hi-kiong, or hayssuen, known to us by the name of Hyson, so called after an East-India merchant of that name, who first imported it into Europe; and the Singlo or Songlo, which name it receives from the place where it is cultivated. Of the bohea teas, the soochuur or sutchong, by the Chinese called Saaty-ang, and Saet-chaon or Su-tyann; the Camho, or Soumlo, called after the name of the place where it is gathered; the cong-fou, congo, or bong-fo; the pekao, pecko, or pehoe; and the common bohea, called Moji by the Chinese. The doctor mentioned other kinds of tea, which were rolled up in the form of balls and threads. He said he had formerly infused all the sorts of green and bohea teas he could procure, and expanded the different leaves on paper, to compare their respective size and texture, intending thereby to discover their age. He found, the leaves of green tea as large as those of bohea, and nearly as fibrous, which led him to suspect that the difference did not so much depend upon the age as upon the other circumstances.

The Asiatics give a flavor to tea by introducing among it the olea fragrans, whose small flowers are frequently to be met with in teas exported from China. On the subject of drinking tea, Dr. L. observed, that the Chinese and Japanese never use tea before it has been kept a year, by which time its narcotic properties are diminished. They drink it without sugar or milk. Having mentioned various methods of preserving the seeds for vegetation, the lecturer entered upon its medical history.

It is natural to conclude, that, as tea was imported from a foreign country, and at no inconsiderable danger and expense, and the custom of drinking it almost universal, much attention would have been excited respecting its natural and medical history, as well as its commercial influence; and, indeed, as the learned president noticed, if saying much is a proof of attention, much has certainly been said and written, and much to no purpose, on its medicinal properties; for, although he has examined nearly one hundred authors on the subject, he has acquired little information, nor can it be expected, when vague hypotheses are substituted for experiment, and theories for facts; thus, claiming no fixed data, the inductions are fallacious or indecisive. This induced the doctor to institute experiments, and establish principles upon which reason might exercise judgment, and truth elucidate facts. From these experiments, which we are sorry our pages will not allow us room to relate, the sedative and relaxing effects of tea appear greatly to depend upon an odorous fragrant principle, which abounds most in green tea, particularly that which is most highly flavored. This seems further confirmed by the practice of the Chinese, who avoid using this plant till it has been kept at least twelve months, as they find, when recent, it possesses a soporiferous and intoxicating quality.

The author deprecated the practice of taking tea very hot, and quoted, in support of his opinion, a passage from Professor Kalm's Travels into North America. The doctor concluded by the following observations: "From the result of the experiments, we may clearly explain the causes of those different effects produced by tea drinking, as well as upon what predominant quality of this exotic these effects depend. Hence it will be inferred, that, when the fine green teas are employed, whose sedative counterbalance their astringent qualities, and particularly in weak or delicate constitutions, debilitating and injurious effects may succeed, as tremors, fluttering and agitation of spirits, pain of the stomach, and weakened digestion, with flatulence, head-ach, and various nervous affections; and, with such constitutions, this tea, taken in the evening, causes watching, and the unhappy sensations which want of the refreshment of sleep naturally produce. And, may it not also be suspected that the increased frequency of palsies and apoplexies, may, in some measure, be attributed to the fragrant, odorous, and sedative, influence of this exotic? Indeed, from the whole analysis of green and bohea teas, the sedative and exhilarating qualities of the former will be clearly comprehended, as well as the astringent qualities of both: although, from the larger proportion of tannin in the bohea, it will be less relaxing, nevertheless combining such a proportion of odor as to give it a grateful influence on the nervous system; and thus, either single or mixed, they convey a pleasant and reviving sensation, as has been so often mentioned by travellers; and persons after fatigue of body, as well as exertion of mind, find in tea a grateful sedative and pleasing diluent."

---

*Medical Society of London.*—The Anniversary of this Society was celebrated on the 8th of last month, when the following gentlemen were returned officers and members of the council for the year ensuing, viz.

*President.*—J. C. Lettsom, M. and LL.D. &c.

*Vice-Presidents.*—Dr. Temple, Dr. Walshman, Mr. Norris, and Mr. Taunton.

*Treasurer.*—Mr. Andree.

*Librarian.*—Dr. Hancock.

*Secretaries.*—Dr. Adams and Dr. Fothergill.

*Secretary for Foreign Correspondence.*—Dr. Taylor.

*Registrar.*—Mr. T. Pettigrew.

*Council.*—Drs. Pinckard, Clutterbuck, Considen, Petch, Lidderdale.—Messrs. Jackson, B. Atkinson, E. Leese, Royston, A. T. Thompson, Blegborough, Bryant, Matthias, Hopkins, Abernethy, Brougham, Sawrey, Adams, Platt, Harvey, Bartlett, Combe, Harding, Sutliffe, Powell, Stevenson, W. R. Griffith, E. Austin, G. Young, Morgan.

To deliver the Anniversary Oration, 1814, Dr. George Rees.

After the ballot, Mr. Saumarez delivered the Annual Oration, on the principles of Physiological and Physical Science, which was numerously attended.

We abstain, at present, from submitting an abstract of the Oration, as we understand it is to be printed.

---

At the Annual Meeting of the Medical and Chirurgical Society of London, held on the 1st of March, the following gentlemen were elected officers and council for the ensuing year:

President, Sir Gilbert Blane, bart. M.D. F.R.S.—John Abernethy, esq. F.R.S.; Matthew Baillie, M.D. F.R.S.; Thomas Bateman, M.D. F.L.S. Librarian; Robert Bree, M.D. F.R.S. Vice-President; Thomas Chevalier, esq. F.L.S. Vice-President; Henry Cline, esq. F.R.S.; Astley Cooper, esq. F.R.S. Vice-President and Treasurer; James Curry, M.D. F.A.S.; Sir Walter Farquhar, bart. M.D.; Sir Henry Halford, bart. M.D. F.RS.; Robert Gooch, M.D.; William Lawrence, esq. Secretary; Alex. Marcet, M.D. F.R.S. Vice-President; Samuel Merriman, M.D.; C. R. Pemberton, M.D. F.R.S.; P. M. Roget, M.D. Secretary; William Saunders, M.D. F.R.S.; James Wilson, esq. F.R.S.; John Yelloly, M.D. Treasurer; George William Young, esq.

---

On the 15th of February died Thomas Ramsden, esq. in the prime of life. No professional character could be more respected by the superior members of the faculty in the metropolis, nor could any individual dividual be more beloved in a large circle of private friends. Mr. Ramsden was surgeon to Christ's and the Foundling Hospitals, assistant-surgeon to St. Bartholomew's Hospital, and a member of many literary societies; but he will be better known to posterity as the author of a valuable practical work, on the Sclerocele, and other morbid enlargements of the Testicle; and on the cause and cure of the acute, the spurious, and the chronic, Hydrocele. Of this useful volume a very full analysis is given in our Journal (vol. xxv. p. 175). The novel views of the subject which this volume presented are there mentioned with a praise which we still think they deserve, and which we again repeat as a tribute of respect and esteem to the memory of a most excellent surgeon and amiable man.

*Mortality by Small-pox from the Weekly Bills.*

| | | |
|---|---|---|
| February | 16, . . . | 22 |
| | 23, . . . | 20 |
| March | 2, . . . | 18 |
| | 9, . . . | 11 |
| | | 71 |

*Subscriptions received from the Country Practitioners who are of the Community of Associated Apothecaries and Surgeon-Apothecaries of England and Wales.*

(Continued from p. 259.)

*Richmond.*

By C. Bowes, esq.

Bowes, C. Richmond
Bowes, T. Reeth
Caggill, Dimsdale Scorton
Campbell and Brown, Bedell
Dowthwaite, Richmond
Edmondson, Middleham
Hodgson, Darlington
Lancaster, Richmond
Lamb, T. Middleham
Lumb, I. Aldbrough
Theakston, Bedale
Terry, Leyburn

Ernest, Sheffield
Moore, Doncaster
Walker, R. esq. Ridings
Willis, Scarborough
Wilson and Travis, ditto.

*Huddersfield.*

By G. Robinson, esq.

Atkinson, Huddersfield
Bradshaw, ditto
Bennett, Almondbury
Dean, Slaithwaite
Green, Mirfield
Houghton, Huddersfield
Martin, Holmfrith
Newhouse, Huddersfield
Robinson, Huddersfield
Roberts, ditto
Stocks, Holmfrith
Tucker, South Devon Militia
Trotter, Holmfrith
Taylor, Mirfield
Wilks, ditto
Wrigley, Huddersfield.

*Leeds.*

W. Hey, esq. in the Chair.

Messrs. Hey, Brook, Chorley, Cass, Dickinson, Moxon, Stansfield, Teale, Taylor, Wade, Wilson.

These Gentlemen form a Committee to carry certain Resolutions into effect for that District; but the Names of Subscribers are not yet received.

*Durham.*

Alcock, Stockton
Bates, Walsingham.

*Sheffield District.*

Wm. Staniforth, esq. in the Chair.

Messrs. Staniforth, Frith, Sterndale, Shaw, Bower, Favell, Snefford, Jackson, Overend, Moorhouse, Foulds, Staniforth, jun. Shaw, jun. Jackson.

CUMBERLAND.

CUMBERLAND.

*A Meeting held at Carlisle.*

T. Blamire, esq. in the Chair.

Resolutions received, but not the Names of the Subscribers.

WALES.

CARDIGANSHIRE.

*Aberystwith.*

Hughes, Aberystwith
Jones, Wern Newidd.
Noott, Cardigan
Williams, M.D. Aberystwith
Williams, ditto.

GLAMORGAN.

*Swansea.*

By Leyson Rees, esq.

Braine, Swansea
Collins and Rees, ditto
Davies, ditto
Long, ditto
Silvester, ditto

Pendrill, Brecon
Williams, ditto

Hawkins, Monmouth
Morgan, St. David's, Pembroke
Williams, Caermarthen.

NOTTINGHAMSHIRE.

*Retford.*

By I. Hartshorne, esq.

Cavie, Retford
Flower, ditto
Hartshorne, ditto
Holmes, ditto
Russell, ditto

Oldknow, Nottingham.

CAMBRIDGESHIRE.

*Wisbeach Medical Society.*

Butter, Hadenham
Knowles, Soham
Muriel, Ely
Norton, Newmarket
Sandiver, ditto
Stevens, Ely
Swinton, Soham
Spencer, Chippenham
Wilkinson, Long Sutton.

SALOP.

*Broseley.*

By W. Thursfield, esq.

Brookes, Wenlock
Fifield, Broseley
Green, Wenlock
Thursfield, Broseley
Wyke, ditto
Brookes, Much Wenlock
Crumpton, Munslow near Wenlock
Crump and Bidwell, Albrighton
Yonge, W. J. esq. Shefnall.

LINCOLNSHIRE.

*Spalding.*

By W. Vise, esq.

Benson, Holbeach
Dawson, Spalding
Hood, Holbeach
Jenning, Spalding
Vise, ditto
Vise, Holbeach

Bissell, Grantham
Gozma, ditto
Jackson, ditto

*Lincoln.*

By H. Swan, esq. in the Chair.

Franklyn, Lincoln
Hare, ditto
Hett, ditto
Snow, J. ditto
Swan, H. ditto
Swan, H. ditto.

NORTHAMPTONSHIRE.

*Northampton.*

H. Locock, esq. in the Chair.

Clarke, Northampton
Cooke, ditto
Hall, ditto
Locock, ditto
Osborn, Messrs. ditto
Percival, ditto
Rudsdell, ditto
Thomas, ditto
Dix, Long Buckby
Gibbons, Kettering
Outlaw, Wellingbro'
Peck, ditto
Porter, Peterborough.

HUNTINGDONSHIRE.

Smyth, Ramsey
Wilson, Huntingdon.

GLOUCESTERSHIRE.

Welch, Stow.

LANCASHIRE.

*Lancaster.*

I. Baxendale, esq. in the Chair.

Anderton, Lancaster
Baxendale, ditto
Carter, ditto
Edmundson, ditto
Greenwood, ditto

Howett,

Howett, Lancaster
Smith, ditto
Latham, Wavertree
Parkinson and Knipe, Kirkham
Wynne, Liverpool.

*Bolton-le-Moor.*
By Messrs. Moore & Rainsforth.
Subscription for the Practitioners at that place.
(No Names specified.)

*Liverpool.*
— Shuttleworth, esq. in the Chair.
At a very numerous meeting, Resolutions passed, but no Names received.

NORFOLK.

Adcock, Walsingham
Bailey, Thetford
Banks, Holt
Burman and Burt, Diss
Hardham, Wells
Mines, Diss
Sharman, ditto
Sharpe, North Walsham.

CHESHIRE.

*Macclesfield.*
By F. Newbold, esq.
Cockson, Macclesfield
Garrett, ditto
Newbold and Dickenson, ditto
Robarts, ditto
Stone, ditto.

OXFORDSHIRE.

*Oxford.*
J. Bull, esq. in the Chair.
Bull, Oxford
Passand, ditto
Rawlins, ditto
Sandell, ditto
Symonds, ditto
Stone, ditto
Salisbury, ditto
West, ditto
Walker, ditto
Wentworth, ditto
Haynes, J. Chipping Norton
Haynes, C. ditto.

LEICESTERSHIRE.

*Hinckley.*

I. Hill, esq. in the Chair.

Bond, Hinckley

Bucknell, ditto

Bloram, ditto

Hill, ditto

Power, ditto

*Leicester.*
By W. Ludlam, esq.
Arnold, Dr. Leicester
Bishop, ditto
Ludlam, ditto
Morris, ditto
Peake, ditto
Paget, ditto.

WARWICKSHIRE.

Bloram, Alcester
Purton, ditto
Jones, ditto
Fosbrooke, Bidford
Bennett, Tamworth.

WORCESTERSHIRE.

Jukes and Watson, Stourport.

*Stourbridge.*
By W. Evans, esq.
Causer, sen. Stourbridge
Causer, jun. ditto
Dixon, ditto
Evans, ditto
Freer, ditto.

DERBYSHIRE.

*County Meeting.*—T. Haden, esq. in the Chair.
Allwood, Allport
Beaumont, Melbourne
Bowyer, Brailsford
Chawner, Melbourne
Coleman, ditto
Cade, Spondam
Chawner, Breeston
Davenport, Derby
Evans, Belpar
Elam, Chesterfield
Fox, Dr. Derby
Froggatt, Eyam
Godwin, Derby
Green, Chapel en le Frith
Haden, C. T. Derby
Haden, T. ditto
Harris, Shipley
Harland, Ashbourn
Ley, Derby
Lomas, Belpar
Melland, Yeograve
Norman, Ilkeston
Poyzer, Wicksworth
Spencer, Duffield
Smith, Stavely
Taberer, Repton
Valentine, Bolsover
Wright, Derby
Webster, ditto.

SUFFOLK.

*County Meeting at Bury St. Edmunds.*

J. Harrington, esq. in the Chair.

Atherston, Brandon
Barker, Ixworth
Bailey, Thetford
Creed, Bury
Chinery, ditto
Dalton, Hawstead
Gedge, Mildenhall
Harrington, Hartest
Hubbard, Bury
Primrose, Mildenhall
Smith, Bury
Webber, Hopton.

*East Suffolk District, at Halesworth Meeting.*

W. H. Crowfoot, esq. in the Chair.

Able, Halesworth
Brittell, Bungay
Crowfoot, W. M. Beccles
Crowfoot, W. H. ditto
Camell, Bungay
Davey, Beccles
Dashwood, ditto
Davie, Bungay
Freeman, Saxmundham
Haward, Walpole
King, Saxmundham
Last, Stradbrook
Lewis, Bungay
Mayhew, Stradbrook
Primrose, Wrentham
Revans, Halesworth
Symonds, Saxmundham
Sutherland, Southwold
Walker, Walpole.

ESSEX.

*Ilford District.*

W. Cooke, esq. in the Chair.

Andrews, Romford
Cooke, Plaistow
Carruthers, Romford
Davis, ditto
Desormeaux, Barking
Hicks, Bow
Ireland, Barking
May, ditto
Mant, Stratford
Moore, Ilford
Raven, Rumford
Staines, Stratford
Wallis, Bow
Weld, Romford.

*Rochford District.*

Final Resolutions or Subscriptions not yet received.

*Eastern District, at Colchester.*

P. Gretton, esq. in the Chair.

Baker, Malden
Bailey, Harwich
Barker, Colchester
Brewster, Tolleshunt D'Arcy
Carwardine, Colchester
Dixon, Witham
Fox, Dedham
Foakers, Thorpe
Gretton, Colchester
Godfrey, ditto
Holmestead, ditto
Harrison, ditto
Logan, Harwich
Mason, St. Osith
M'Carthy, sen.
M'Carthy, jun.
Nunn and Silke, Manningtree
Nunn, Colchester
Newell, ditto
Perkins, ditto
Patmore, Kelvedon
Parker, Colchester
Rogers, ditto
Rogers and Co. Manningtree
Smith, Colchester
Smith, Wivenhoe
Salter, Boxford
Smith, Tolleshunt D'Arcy
Tweed, J. Braintree
Taylor, Earl's Colne
Travis, jun.
Tweed, R. Braintree
Wiltshire, Weathersfield

Armstrong, Sible Hedingham
Eachus and Son, Saffron Walden
Gilson, Halstead
Griffen, Epping
Godfrey, Coggeshall
Harrison, Braintree
Holmstead, Bocking
Kelvedon, Frost
Lynch, Dunmow
Pearson, ditto
Welch, Stanstead Mount Fidget
Walker, Ongar.

HEREFORD.

Resolutions received, but not the Names of Subscribers.

CORNWALL.

Burgess, Loswithiel

Lanyon, ditto.

MIDDLESEX.

Brand, A. E. esq. Chiswick
Broxholm, Sunbury

Bunnett

Bunnett and Son, Fulham
Douglas, sen. Ealing
Day, Isleworth
Gillman, Highgate
Griffenhoofe & Sheppard, Hampton
Hyatt, Ealing
Monson, Tottenham
— Radford, esq. Hammersmith
Snow, Highgate
Toulmin and Co. Hackney.

SURRY.

*County Meeting, at Kingston.*

T. Parrott, esq. in the Chair.

Brown, C. Cobham
Brown, I. Esher
Baker, Thames Ditton
Fletcher, Croydon
Gapper, Ewell
Hardwick, Epsom
Jackson, Guildford
Martin, Ryegate
Maud, Epsom
Montague, ditto
Parrott, Mitcham
Sandford, Wimbledon
Steele, Ryegate
Sharpe, Messrs. Wandsworth
Smith, Crawley, Sussex
Taylor, Kingston
Wallace, Carshalton

Andrews, Richmond
Ansell and Curtis, Dorking
Bottomley, Croydon
Baker, Leatherhead
Chapman, Wandsworth
Dalton, Dorking
Edwards, Putney
Gardner, Clapham
Irish, Frimley, near Bagshot
James, Croydon
Julius, Richmond
King, Mortlake
Mallett, Dorking
Newnham, Farnham
Parrott, jun. Tooting
Phillips, Carshalton
Smart, Wandsworth
Sharpe, Tooting
Shillito, Putney.

*Rotherhithe.*

By W. Gaitskell, esq.

Brown and Randell, Rotherhithe
Ford, ditto
Gaitskell, ditto
Wright, ditto.

BEDFORD.

*County Meeting, at Ampthill.*

Charles Short, esq. in the Chair.

Chapman, T. Ampthill
Chase, Luton
Chapman, C. ditto
Dyer, ditto
Gibbon, Bedford
Green, Woburn
Gall and Son, Biggleswade
Layman, Snefford
Lawford, Leighton Buzzard
M'Grath, Biggleswade
Pain, Harrold
Pulley, Bedford
Penrose,
Parker, Woburn
Small,
Short, Bedford
Verrall and Payne, Potton
Wagstaff, Leighton Buzzard
Farr, Dunstable
Wingfield, Market-street.

SUSSEX.

*Rye District.*

By J. Bellingham, esq.

Bellingham, Rye
Butler, ditto
Bishop, T. Tenterden
Bishop, W. ditto
Hougham, Brookland
Lewis, New Romney
Mace, Tenterden
Ramsden, Rye
Satterly, Hastings
Sutton, Winchelsea
Wellings, Hastings
Walter, New Romney
Wilmanton, ditto
Young, Hawkhurst.

*Brighton.*

By I. Hall, esq.

Bond, Brighton
Brewster, ditto
Barrett, ditto
Blaken, ditto
Batcock, ditto
Hall, ditto
Newnham, ditto

Bethune, Brighton
Crockford and Roberts, Lewes
Colgate, East Hoathley
Hodson and Bellingham, Lewes
Hill, Arundel
Ingram, I. Steyning
Ingram, H. ditto

Lane, Arundel
Rickwood and Son, Horsham
Stone, Mayfield
Woodman, Bognor
Whicher, Petworth
Weeks and Son, Hurst per Point
Wheeler, Little Hampton.

WILTSHIRE.

*County Meeting, at Salisbury.*
H. Smith, esq. in the Chair.

Andrews, Salisbury
Beckingsale, ditto
Cave, ditto
Coates, ditto
Fisher, ditto
Gutch, Wallop
Hooper, Downton
Nunn, White Parish
Pargiter, Fordingbridge
Smith, Salisbury
Winzar, ditto
Wells, ditto
Whitmarsh, G. Downton
Whitmarsh, R.

*Highworth District.*

By R. Marsh, esq.

Marsh, Highworth
Mantell, Farringdon
Skinneier, Archlade
Slayter, Fairford
Wills, Cricklade
Williams, Swindon
Macfarlane, Devizes.

DORSETSHIRE.

*County Meeting, at Dorchester.*
R. Bever, esq. in the Chair.

Arden, Dorchester
Bever, Weymouth
Basket, Blandford
Cooper, Weymouth
Druitt and Son, Winborne
Daniel, Beaminster
Downe, Bridport
Dansey, Blandford
Ellis, Weymouth
Edwards, Dorchester
Everingham, Wareham
Grey
Highmore
Hodges, Mardon
Hingestor, Lyme
Lamb, Beaminster
Moul, Weymouth
Miller, Poole
Roe, Winborne
Robinson and Son, Bridport
Stains, Wareham
Smart, Cranborne
Turner
Tucker, Lyme
Trowbridge, jun. Cerne
Worne, Weymouth
West, Poole
Wallis.

*Cerne.*
By Messrs. Stein and Davis.
Six subscriptions for Cerne and Sherborne. Names not received.

HAMPSHIRE.

*County Meeting.*—W. N. Wickham, esq. in the Chair.

Wickham, Winchester
Lyford, ditto
Hewlitt, ditto
Mayo, ditto
Seeds, Portsea
Wickham, Sutton Scotney
Nike, Lymington
Towsey, ditto
Blunden, Botley
Rogers, Westmeon
Davies, Andover
Crabb, Romsey
Coleman, ditto
Beddum, ditto
Keale, Southampton
Keale, J. R. ditto
Barnard, ditto
Maul, ditto
Corfe, ditto
Ready, ditto
Cole, Whitchurch
Hedden, Broughton
Harpur, Gosport
Soaper, Southwark
Seymour, Bishop's Waltham
Downer, Milbrook
Whicher, Petersfield
Woolley, ditto.

*Havant.*
By J. Hicks, esq.

Hicks, Havant
Bannister and Son, ditto
Hicks, Emsworth
Hogg, ditto.

HERTS.

Churchill, Hitchen
Collier, Berkhempstead
Forster, ditto
Smith, Great Haddam
Scarr, Bishop's Stortford
Lloyd, Tring
Firth, ditto

 Kilby,

Kilby, Watford
Masters, ditto
Paumier, ditto
Lucas, Hatfield
Stewart, ditto
Jones, Stevenage
Harrold, Cheshunt
Bricknell, Sawbridgeworth.

BUCKS.

Rumsey, Dr. Amersham

Rumsey, J. ditto

Hayward, Aylesbury

Terry, ditto.

BERKS.

*Reading District.*

Ring, Bulley, and Co. Reading
Hooper and Sherwood, ditto
Golding, ditto
Smith, Whitchurch
Newbolt & Wheeler, Oakingham
Kidgell and Trew, Pangborne.

*Windsor District.*

By ——— Chapman, esq.

Battiscombe, Windsor
Thomas, ditto
Chapman, ditto
O'Reilly, ditto
Wilmot, Eton
Ives, Chertsey
Summers, ditto
Earle, ditto
Furnival, Egham
Lindeman, ditto
Bingham, Uxbridge
James, ditto
Rayner, ditto
Wall, Maidenhead
Robarts, Burnham
Lamb, Dr. Newbury
West, Abingdon
Bishop, Maidenhead
Taylor, Wargrave
Somerset, Lambourne
Cork, ditto
Wheeler, Wokingham.

KENT.

*Dover.*

By Stephen Chalk, esq.

Thatcher, Dover
Norwood, ditto
Elsted, ditto
Chalk, ditto.

*Deal.*

By W. Hulke, esq.

Hulke, Deal
Snowden, ditto
Hougham, ditto
Fitzgerald, ditto
Day, Maidstone
Charles, W. ditto
Charles, T. ditto
Haffender, ditto
Knight, T. ditto
Knight, Folkstone
Baildon, Deptford
Giraud, Faversham
Lukyn and Wildash, ditto
Perfect & Ralph, Town Malling
Sutton, ditto
Watson, Staplehurst
Carruthers, Tunbridge
Pullen & Mogg, Tunbridge Wells
Pout, Yalding
Pharmacopola Verus, C.
Taynton, Bromley.

---

*An Address, explanatory of the Motives of the present Application to Parliament by the Apothecaries and Surgeon-Apothecaries.*

A Bill having been introduced into Parliament, "for regulating the Practice of Apothecaries, Surgeon-Apothecaries, and Practitioners of Midwifery, and of all Compounders and Dispensers of Medicines throughout England and Wales;" the Committee, who were appointed by a General Meeting of Apothecaries convened by public advertisement, and authorised to adopt such measures as should appear best calculated for securing the above objects, feel it an incumbent duty to publish a very brief statement of their views.

In doing this, they must remark, that every branch of the medical profession, liberally and scientifically practised, is an honorable and useful occupation; and that the public good demands that it should be exercised upon such principles.

Apothecaries and surgeon-apothecaries are the most numerous class of of medical practitioners: their duties are of very serious and universal interest, the lives and health of by far the greater part of the community being entirely confided to their care, without in many instances the possibility of obtaining other advice; yet they are allowed to practise without any examination or test of their competence whatever; so that any person, however destitute of medical or even common education, may assume with impunity the character and functions of the apothecary. This is a great source of evil both to society and themselves, and the necessity of preventing it by the establishment of adequate regulations is sufficiently obvious, and universally allowed. The Royal College of Physicians have acknowledged it, by having recently suggested a plan for its attainment.

Such an order of things could certainly never have existed were it not for the extreme difficulty of judging of the real merits of medical men, which exposes the public to the imposition of pretenders, whose ignorance and confidence are generally commensurate. Hence it is surely of the highest importance that there should be some authority for examining and deciding upon the qualifications of all persons who may hereafter intend to practise as apothecaries, surgeon-apothecaries, or midwives, or to act as compounders and dispensers of medicines.

The Committee with deference conceive that apothecaries and surgeon-apothecaries are entitled to additional regard from the consideration, that the numerous medical practitioners required for the service of the army, navy, merchants' ships, and our very extensive colonies, chiefly derive the rudiments of their education from those branches of the profession. It is notorious, that the public service has frequently been distressed from the small number and inefficacy of medical persons appointed, in consequence of few comparatively being now regularly educated under apothecaries.

Another subject of complaint is the unfortunate mode of remuneration which custom has established—a mode which is repugnant to the feelings of every liberal and conscientious mind, and equally prejudicial to the interest of the patient and that of the practitioner. In proposing a different method, the Committee are fully aware that moderation will best insure the countenance of the public; and therefore suggest that the present charge for medicines shall be reduced, and that apothecaries in future shall possess a legal claim to receive a fair compensation for their professional skill and attendance. They sincerely hope to see this plan gradually adopted, confident that its advantages will be reciprocal. It is not desired that their attendance should be made more expensive; the contrary will often be the case. It is the mode, and not the amount of remuneration to which they have cause to object.

The proposed manner of ascertaining the competence, and of remunerating the services of the apothecary, and an arrangement for medical attendance upon parochial poor, which has long and loudly called for legislative interference, are the important objects to which the attention of parliament is solicited.

The Committee are persuaded that the good of the public and the respectability of their branch of the profession are intimately connected, and attainable by the same means; in the pursuit of which they have sedulously and respectfully endeavored to obtain the concurrence of the Royal College of Physicians, the Royal College of Surgeons, and of the Society of Apothecaries, upon whose rights, powers, or privileges, it is well known they have no desire to encroach. The authority of the Royal College of Surgeons, as well as that of the Society of Apothecaries, embraces only the members of their own respective bodies, and does not take cognizance of their professional qualifications as practitioners of medicine, while the jurisdiction of each of the above bodies is confined within a circumference of ten miles round London.

A very extensive correspondence with several of the most intelligent and respectable practitioners in every county in the kingdom, has fully proved their general approval of the measures in question: they, as well as those of London, are sincerely anxious to have such provisions enacted as will promote the welfare of the community, while they tend to lessen the embarrassments which so sensibly affect the character and interests of the profession.

The Committee beg leave to express an implicit confidence that the object and intention, duly and impartially considered, will justify their expectation of universal approbation and support.

By desire of the Committee,

*London, March* 11, 1813. G. M. BURROWS, Chairman.

*Outlines of a Plan proposed by the London Committee, for Improving and Protecting the Profession of the Apothecary, Surgeon-Apothecary, and Practitioner in Midwifery, in England and Wales.*

I. The provisions of the proposed bill, so far as relates to qualifications, will be prospective, and therefore not applicable to persons already in practice.

II. No person to be allowed to enter the profession without having served a regular apprenticeship, or producing testimonials of a suitable medical education.

III. No person to be allowed to practise as apothecary, surgeon-apothecary, or accoucheur, without obtaining a certificate after due examination of his qualifications.

IV. No person to be allowed to practise in any branch of the profession, or to compound or dispense medicines, without taking out an annual license.

V. Every indenture of apprenticeship to bear a stamp duty, not exceeding 25*l*.

VI. A superintending body to be legally constituted for the several objects of the proposed act, with power to make such by-laws and regulations as may be necessary to carry them into effect.

VII. England and Wales to be divided into a certain number of medical districts, of which London shall be the superior, and where only certificates for qualifications can be obtained.

VIII. Assistants to undergo an examination.

IX. There shall be a legal right for apothecaries, surgeon-apothecaries, and accoucheurs, to claim a moderate charge for attendance, as visits or journeys, that for medicines being reduced.

X. Regulations

X. Regulations for attendance upon parish poor.

XI. A register to be established in every district of all licensed practitioners, apprentices, &c.

THE COMMITTEE.

Mr. Burrows, Chairman, Bloomsbury-square
— Simons, Soho-square
— Chilver, New Burlington-street
— Brande, Arlington-street
— Walker, St. James's-street
— Tegart, Pall Mall
— Field, Christ's Hospital
— Upton, Throgmorton-street
— Hunter, Mincing-lane
— Rivers, Spring Gardens
— Hurlock, St. Paul's Church Yard
— Wells, Ely-place
— Ewbank, Upper Grosvenor-street
— Pennington, Montague-place
— Forbes, New-street, Hanover-square
— Douglas, Bedford-street, Bedford-square
— De Bruyn, North Audley-street
— Cates, Argyll-street
— Drew, Gower-street
— Brown, Raven-row, Spitalfields
Mr. Pilliner, Dartmouth-street
— Bowman, Harley-street
— Yonge, Bennet-st. St. James's
— Jones, Mount-street
— Bamford, Conduit-street
— Hobson, Great Mary-le-bone-street
— Harkness, Ratcliff
— Fernandez, New Ormond-street
— Good, Guilford-street
— Thomson, Sloane-street
— Atkinson, Jermyn-street
— Parkinson, Hoxton-square
— Kerrison, New Burlington-street
— Royston, Princes'-street, Cavendish-square

*Honorary Members.*

Mr. Nunn, Colchester
— Gretton, ditto
— Griffinhoofe, Hampton
— Shillito, Putney
— Bliss, Hampstead
— Cook, Plaistow, Essex
— Julius, Richmond
— Gardener, Clapham.

---

The London Committee having met the Deputies of the County Districts, (who came from the most remote parts of the kingdom) on Tuesday the 23d instant, at the Crown and Anchor; and, on Wednesday the 24th, a General Meeting of the London practitioners being convened at the same place; so many new views of the subject were developed, which promised so much improvement to the plan, that the Committee has thought it expedient to withdraw the Bill for the present session, with the avowed intention of submitting it to parliament next year, under circumstances of considerable melioration.

---

Mr. James Ward, R.A. has in the press, Some Account of Mary Thomas and Ann Moore, the two Fasting Women; embellished with etchings, executed in the best manner of the art. This is intended to be an imperial folio, with seven etchings, consisting of portraits and illustrative views.

---

Dr. Merriman's Spring Course of Lectures, on the Theory and Practice of Midwifery, and the Diseases of Women and Children, will commence on Monday, April 5, 1813, at the Middlesex Hospital; when he will deliver an introductory lecture, on the present State of the Practice of Midwifery in England.

METEO-

